# The Hospital. Nursing Section

**Published:** 1905-01-07

**Authors:** 


### The Hospital.

### Nursing Section.

Contributions for this Section of "THE HOSPITAL" should be addressed to the EDITOR, "THE HOSPITAL," NURSING SECTION, 28 & 29 Southampton Street, Strand, London, W.C.

No. 954.—Vol. XXXVII. *SATURDAY, JANUARY 7, 1905.*

### Notes on News from the Nursing World.

### THE ORIGINATOR OF THE INDIAN NURSING SERVICE.

PIONEERS are so often forgotten by people with short memories that, at a time when the Indian Army Nursing Service is attracting to its ranks several of our valued nurses at home, including one matron of a London hospital who has just joined, it seems only fair that credit should be given to the persevering lady who was the originator of the movement. To supply trained nurses for the military hospitals in India was a scheme which Lady Roberts had much at heart when she was residing in that country, and as far back as 1886 she sent as an experiment the nurses belonging to her nursing staff to work in the station hospitals at Meerut, Lucknow, Bareilly, Quetta, and other places where a large number of troops were stationed. These nurses had great difficulties to contend with, the nursing having hitherto been done by private soldiers, who, no matter how willing and kindhearted, were necessarily ignorant and untrained. But the work done by these pioneers proved so successful that Lady Roberts was encouraged to ask the help of the Secretary of State. Her recommendations were favourably received, and he undertook to despatch nurses at Government expense. Now every large military hospital in India has its staff of trained nurses, and there are special wards provided for the officers. Parents who are parting from their sons who have to join their regiments in India may feel confident that during times of sickness and disease their dear ones will have the benefit of good nursing and proper attention, which in former years it was so difficult to obtain.

### HOMES FOR NURSES IN INDIA.

ONE of the conditions made by the Secretary of State in acceding to the request of Lady Roberts was that she should provide homes in the hills where the nurses could go when on furlough or on sick leave, a stipulation to which she readily assented. The best known home for the nurses is the one at Murree, where a great many of the Army nurses have stayed during their holidays and when requiring rest and change. Murree is situated on one of the lower ranges of the Himalayas, where it has been said that Dame Nature has been more lavish of her charms than in any part of the world. It is about 40 miles from Rawalpindi, and to get there one has to take a tonga journey of about six hours. Nurses arriving from the heat of the plains never fail to enjoy the fresh mountain air and the lovely scenery.

### THE CASE AGAINST CUBICLES.

THE chairman of Grimsby and District Hospital, observing our reference to Cheltenham Hospital a fortnight ago, asks us why we consider cubicles "a serious drawback." His reason is that it is proposed to provide cubicles in the new home for the nurses of Grimsby Hospital, now in course of erection, and the chairman, like a wise man, desires to prevent any avoidable mistake in construction, if cause can be shown for a change of procedure. He postulates, however, the argument that because cubicles give a free circulation, they are more healthy than separate rooms. We cannot see how a room in which many sleep, and where there is a free circulation of air above the cubicles only can be more healthy than a smaller room in which but one person sleeps. A nurse who has a bad cough, or who snores—if a nurse ever snores—necessarily disturbs the sleepers in cubicles, whereas if she occupies a bedroom she is not a nuisance to any one. In cubicles it is possible for nurses who are not particularly tired to keep some of their fellow-workers awake who are weary enough to desire to sleep at once; and influenza and colds spread far more quickly among the staff when they sleep in cubicles than when each one has a room to herself. It is the experience of the matrons of nearly all our great hospitals that single rooms, with proper ventilation, are perfectly healthy, and we do not know of one who would go back to the cubicles which, in many instances, they have displaced. But the primary argument in favour of single bedrooms as against cubicles is that it secures for a nurse the only real privacy she is likely to obtain while she is in hospital. If she has a bedroom to herself it is as much her castle as the Englishman's house, and she ought to have it wherever the accommodation is possible. The chairman of Grimsby Hospital, who shows such kindly anxiety to consider the interests of the nursing staff, admits the possibility in the case of that institution; and we assure him that if he wishes to attract the best class of probationers and make them as comfortable as he can, he will propose the revision of the plans for the home and cut out the cubicles.

### MANCHESTER CORPORATION AND THE MIDWIVES ACT.

AN important step has been taken by the Manchester Corporation, in view of the fact that the Midwives Act comes into force on April 1st. In August last a special committee of 12 members was appointed with the title of the Midwives' Supervising Committee, to whom were delegated the powers of the Council as the local supervising authority. The committee have just recommended Dr. Margaret Mary Smith, at present resident medical officer at Craigleith Poorhouse, Edinburgh, and formerly resident physician at the Mater Infirmorum Hospital, at Belfast, as the "executive officer" under the Act, with a salary of £250. Her duties are very clearly defined. It will be the business of the new "executive officer" under the Manchester Corporation to visit midwives, both systematically and irregularly, in order to see that their homes are kept in a sanitary condition, to examine the cases or bags of instruments of the midwives, to ascertain that their case-books are properly kept, to satisfy herself that they understand the use of antiseptics, and to exercise her supervision so far as to see that the midwives are suitably provided with washable dresses. She will also be required to visit the cases attended by the midwives, in order to assure herself that the requisite procedures are carried out, that strict cleanliness in all things is observed, and that suitable provision is made for the dieting both of mother and child. As this is the first appointment of the kind in the north of England it has excited great interest.

### THE UNTRAINED NURSE IN VICTORIA.

It is noteworthy that, following the letter of Miss Glover on "Voluntary and Compulsory Registration," which appeared in our issue of last week, we have before us a report of a meeting of the Royal Victorian Trained Nurses' Association, at which complaints were made that, in spite of registration, the competition of untrained nurses in the colony is increasing. "I know," said one of the registered nurses who spoke at the meeting, "of three so-called 'gamps' who are at present making an average of £3 per week by visiting nursing," and, she added, "the 'gamp' is not dying out, for every day of my experience brings fresh proof of the numbers that are entering into the running."

### DISTRICT NURSING AND TRADES COUNCILS.

A very interesting incident took place at Swansea the other day, when a deputation of ladies belonging to the District Nursing Institution visited the Working Men's Club for the purpose of enlisting the sympathy of the Trades Council in its work, and in order to induce it to bring its influence to bear upon the workmen of the town to contribute a regular, if small, subscription. Several questions were asked by members of the Council, and ultimately it was decided that arrangements should be made for all the affiliated branches to receive a similar deputation accompanied by a member of the Trades Council. The ladies representing the Nursing Association expressed gratification at their reception, and we hope that the practical result will be a considerable augmentation to the regular income of the organisation. Nothing but good can come from co-operation between district nursing associations and working men, who very often only require such a stimulus as that which seems to have been applied at Swansea in order to afford their steady support.

### THE EMPLOYMENT OF WARDMAIDS AS NURSES.

The Kilrush Guardians, taking a leaf out of the book of the Kidderminster Guardians, have determined to set the Irish Local Government Board at defiance. An inspector of the board, after an exhaustive inquiry into the system of the workhouse hospital and infirmaries, has reported that it is most unsatisfactory. He found that several of the wardmaids were really acting as assistant nurses, contrary to the terms of their employment, and he recommended that the services of six of the wardmaids should be dispensed with and two qualified nurses be appointed. One of the guardians, commenting on the recommendation, said, "We are not going to submit to the doctrine of being mere figureheads here without a mind of our own," and it was ultimately determined to continue the existing nursing arrangements. It now devolves upon the Irish Local Government Board to insist that wardmaids shall not be permitted to perform nursing functions in Poor Law institutions in the sister island.

### AMERICAN NURSES IN CONFERENCE.

A conference of American visiting, health, board, and school nurses was held last month in the library of the New York Charity Organisation Society. About forty-five were present, and such subjects as uniforms, and the care of contagious, obstetrical, and tubercular cases were discussed. It was decided to hold four meetings of a similar character during the year, and to keep the meetings informal, with no constitution or by-laws. The sole object of the conferences is the exchange of news and experiences, and it is obvious that this may be of great practical advantage to those who attend them. In order to defray current expenses each nurse contributes a trifle.

### IMPORTANT APPOINTMENTS TO CAMBERWELL INFIRMARY.

We are officially informed that a number of important appointments have been made to Camberwell Infirmary, consisting of seven new sisters and seven new staff nurses. The sisters are Miss Gertrude Carter, who, having been trained at Guy's Hospital, and at Goole Cottage Hospital, has since been attached to Guy's Nurses' Institution; Miss Mary A. Elton, who was trained and has been sister at the Royal Infirmary, Bradford; Miss Flora M. Freeman, who was trained at Hackney Infirmary; Miss Isabella Phillips, who was trained at Leeds Infirmary, and has since been private nurse at Victoria Hospital, Chelsea; Miss Annie M. Riddell, who was trained at Camberwell Infirmary, and has since been sister at Fulham Infirmary, and private nurse; Miss Jeannie Rodgers, who was trained at Camberwell Infirmary, and has since been charge nurse at Park Hospital, Hither Green; Miss Norah L. Sutcliffe, who was trained and has been staff nurse at the Royal Devon and Exeter Hospital, and has served in South Africa as a sister of the Army Nursing Service Reserve. The staff nurses are Miss Rose Bellamy, who was trained at St. Mary Islington, Infirmary, Highgate Hill; Miss Annie Davis, who was trained at Acton Infirmary; Miss Ellen M. Denyer, who was trained at St. Olave's Infirmary; Miss Emma F. Fay, and Miss Mabel Meech, who were trained at Portsmouth Infirmary; Miss Daisy A. Metcalfe, who was trained at Southwark Infirmary; and Miss Lauretta Sharpe, who was trained at the Central London Sick Asylum, Hendon.

### THE SCOTTISH BRANCH OF THE QUEEN'S JUBILEE INSTITUTE.

According to the sixtieth annual report of the Scottish Branch of Queen Victoria's Jubilee Institute there are now 257 Queen's nurses in Scotland.

The number of branches is 165, six of which were established during 1904. It is only in consequence of inadequate funds that the Council have not provided nurses for all the poorer outlying districts and islands which are in need of them. The financial support given during the twelve months was larger than in the previous year, but it still falls short of the necessary expenditure, and the Council naturally feel that they must not further increase operations until they are assured of an increase of income. One extremely satisfactory feature of the report is that there were 249 contributions on behalf of patients, amounting to £115.

### THE WEST LONDON HOSPITAL.

An extremely interesting account appears in another column of the farewell party given by Miss Irene Hardy, the retiring lady superintendent of the West London Hospital, Hammersmith. The admirable service rendered by Miss Hardy, who for a quarter of a century has been connected with the hospital, and the esteem in which she is held by all members of the staff, past and present, could not have been better demonstrated than by the proceedings on Saturday evening. We join with pleasure in the testimony which was offered on that occasion to her capacity, her devotion, and her successful efforts in elevating the standard of nursing, in the school of which she was for so long the greatly esteemed head. It is satisfactory to know that, in the person of Miss Nevile, who has been Miss Hardy's assistant since 1900, a suitable successor has been appointed, and that in her hands the interests of the institution will be safeguarded.

### DISTRICT NURSING IN JERUSALEM.

It is interesting to learn from Jerusalem that between May 1903 and November 1904, 1,460 district visits were paid by the two nurses from the home in connection with Bishop Blyth's Mission, and that from October 1903 these nurses attended 780 dispensary cases. Owing to one of the nurses having contracted typhoid fever through nursing a case, she was invalided for more than four months, and as her colleague had to attend to her, the work almost ceased. Both nurses, who also treat the minor complaints of the children from the Bishop's day school in Jerusalem, are now on duty again.

### PROGRESS AT SHEERNESS.

Since 1902, when the Sheerness Nursing Society was affiliated to Queen Victoria's Jubilee Institute, it has made rapid strides, including the acquisition of a small house facing the sea, which was purchased at a cost of £490. In 1903 the patients—many of whom were soldiers and sailors—being too numerous for one nurse to attend to, a second was engaged, the Admiralty making an annual grant of £39 towards her support. At the end of that year the committee of the society had the gratification of announcing that they possessed a balance of £60 in hand, and in 1904 two concerts given in aid of the funds yielded a total of £82. In 1902 not a single doctor in the town would identify himself with the society; now the movement has practically the entire sympathy of the medical men.

### THE MANUFACTURE OF COTTAGE NURSES.

At an amateur dramatic entertainment on behalf of the Essex County Cottage Nursing Association Lady Rayleigh said she thought that the only way to make the organisation thoroughly well known would be to hire entire pages of the county paper—and of a London daily paper as well—and print upon them "Essex County Cottage Nursing Association, the best and cheapest home for nurses; give freely and quickly if you would not be too late, and if you care for the health and happiness of the poor of Essex." Perhaps some generous supporter of the organisation may arrange for it to be acted upon. The suggestion could not, she admitted, be carried out, because there was not sufficient capital at the command of the Association. The object of the Essex Association, Lady Rayleigh states, is the manufacture of nurses; the training is only for six months, and those who receive it at Leyton are required to serve the Association for two years at a fixed salary.

### THE MATRON OF GLOUCESTER GENERAL INFIRMARY.

Miss Yeats, on her retirement from the matronship of the Gloucester General Infirmary and Eye Institution, after 17½ years' service, has been presented with a silver inkstand by the medical and surgical staff, and a silver tea service and tea tray by past and present officers, members of the nursing staff and household, in token of their esteem and in recognition of her work amongst them for so many years.

### DEATH OF A MATRON.

We regret to hear of the sudden death of Miss Greenhough Smith on December 27th at the Dial House, Seend, the residence of her sister. Miss Smith was for many years matron of the Bristol Royal Infirmary, and for the past seven years was matron of the Bristol Blind Asylum. She was trained at St. Bartholomew's Hospital where she obtained the gold medal and was afterwards night superintendent. She then became temporary matron of Charing Cross Hospital and afterwards matron of Homerton Fever Hospital. Miss Smith was laid to rest on Friday last in the quiet little churchyard at Seend, a memorial service being held on the same day at All Saints' Church, Bristol. The chaplain of the Bristol Branch of St. Barnabas Guild conducted the service. A large number of friends were present, including members of the Guild and the staff of the Bristol Royal Infirmary. The nursing profession has incurred a real loss by the death of Miss Smith, who was loved by all who knew her.

### MISS MONK ON STATE REGISTRATION.

Owing to the pressure on our space this week we are compelled to hold over an article on Miss Monk's contribution on "State Registration," which appears in the *Monthly Review* of January.

### SHORT ITEMS.

The nursing staff of St. Mary's Infirmary, Highgate Hill, have presented Dr. A. H. Robinson, the medical superintendent, with a handsome revolving mahogany bookcase as evidence of their goodwill, and his wife with beautiful flowers.—The festivities this year in connection with the Weymouth Trained Nurses' Institute were kept on Boxing Day, and a very enjoyable time was spent. The superintendent and matron were the recipients of presents.

### The Nursing Outlook.

"From magnanimity, all fear above;
From nobler recompense, above applause,
Which owes to man's short outlook all its charm."

### NURSES AND SANITATION.

THERE is no doubt that a knowledge of sanitation is a very important matter to the nation, and that one of the best means of spreading such information is by the co-operation of women. Legislation can do much good, but until each woman in her home elects to be her own sanitary inspector very slow progress can be made. The progress of sanitation largely depends upon two points—the administration of sanitary law by sanitary authorities, and the co-operation of the people in interpreting the law. To have grasped the necessary points of good sanitation is of the utmost value to a nurse, because she is essentially a practical teacher. Her influence in a district among the very poor is at all times powerful, and if she thoroughly grasps the principles of sanitation, and does her best to get those around her to carry them out, she is doing a great and important work. It is to our nurses that we must mainly look to give instruction in the simple laws of health in the humblest dwellings; because they can obtain access to homes where legislation is powerless, and teach the evils of a blocked drain, a dirtily kept sink, bad cooking, wrong feeding of babies, overcrowding, and the value of fresh air and good water. There are now at all our large hospitals schools for probationers, and it is in these training schools that the principles of sanitation should be taught.

Sanitation, as a subject, of course covers wide ground, but two points especially are of value to the future nurse. First, as to the drainage of a house. Bad drainage results from bad work and from want of management. Drains require care; otherwise the proverbial "bad smells" come. But these can be made to disappear if the drain-pipes are flushed with clean water.

The water-supply must be considered: the cistern should be covered, it should be easy to get at for cleaning, and so placed as to avoid risks of impurity. In many poor houses there is only one cistern, and the lid of this is generally used as a resting-place for dirty dusters, brooms, and pails, besides other unpleasant arrangements for actually fouling the water-supply. The fact of there being only a single cistern in a house inhabited by a number of families is a common cause of infection, for water is carried up over-night in an uncovered wooden pail, and often left reposing under a bed, or with free access for the children, who dip dirty small hands into it in order to slake their thirst. The nurse with a knowledge of sanitation can do a good deal to remedy these evils. The proper storage of food in the homes of the poor is a weighty matter and a very difficult one to tackle among families who exist in one or two rooms. Obviously, the best place is outside. The area is the next best place if kept in a cleanly condition and with satisfactory drainage; another alternative is a small wire safe hung outside a window. Frequently the food is kept in an old wooden box, which is seldom cleaned out.

The state of Conveniences in such houses is often better imagined than described. They are generally hopelessly dirty, with no proper water-supply, and a never-opened window.

Dustbins badly kept, with food and vegetable refuse thrown in, form a wide field of usefulness to a nurse who understands sanitation, and she will urge that anything which can putrefy or cause infection should be burnt daily in a glowing fire.

Then, as to infection and disinfection, a knowledge of these is indispensable to the nurse to enable her to speak with authority and to tell the people concerned that they have no personal will, but must simply obey the law.

An epidemic of scarlet fever happens. If the nurse is acquainted with the provisions of the Infectious Diseases Notification Act 1889, the Infectious Diseases Prevention Act 1890, and the Isolation Hospitals Act 1893, she can proceed far more vigorously than if she is ignorant of them. It is useful for her to know that the law forbids an infected person appearing in the public streets or entering a public conveyance, that infected articles are disinfected free of charge at the disinfecting station, and that it is unlawful to let for hire a house or room in which a person has recently suffered from an infectious disease, the penalty for disobedience being a fine of £20. According to Art. 88 of the Education Code, approved of by the Board of Education, a sanitary authority has the power, should infection show itself in an elementary school, to either notify to managers that special pupils must not attend for a particular time, or to close the school for a period. But a sanitary authority has no power over Sunday or private schools, and in these cases a nurse who understands the processes of infection can be of material aid to managers.

Finally, it would be wise for all nurses to obtain the certificate of the Sanitary Institute at the expiration of their training in hospital. The syllabus of this examination covers a wide field, and is of a most practical character. The study of sanitation would undoubtedly benefit the nurse in a multitude of ways, and promote the usefulness of her career. It would generally enlarge the scope of her ideas on a subject which is vital to the health and well-being of the poorer sections of the people especially.

### Lectures Upon the Nursing of Mental Diseases.

By Robert Jones, M.D.Lond., B.S., F.R.C.S.Eng., M.R.C.P.Lond., Resident Physician and Superintendent of the London County Asylum, Claybury.

### LECTURE XXIII.

The remainder of the space allotted to us will be taken up with the consideration of accidents and emergencies, which occur most unexpectedly among the insane, in spite of the closest supervision and precautions. With a certain proportion of the inmates of every institution for the insane bent upon self-destruction, 21 per cent. of all cases admitted; with a certain number subject to epileptic seizures without warning, 8 per cent. of all cases admitted; and, further, with some who are violent in the extreme upon the slightest suspicion of actions contrary to their delusions, the occurrence of accidents within an asylum or upon its grounds is not to be wondered at. It is necessary, therefore, that the mental nurse should be in a position to deal promptly with some of these, pending the arrival of the medical officer, who must be summoned at once, and who in these institutions is, fortunately, always somewhere near at hand.

The question of dealing with cases of self-poisoning will be first considered, and there are three leading principles which must be considered for guidance in such emergencies. Primarily, the necessity for getting rid of the poison is obvious, and is best obtained by means of emetics, in regard to which there are certain definite rules to be observed as to their use. They are to be administered always, and at once, when there are no signs of corrosion or burning about the mouth or lips after poison has been swallowed; the safest of these being mustard and warm water, or warm water and salt. When signs of corrosion are present an emetic is not to be given, but oil administered. Secondly, if unable entirely to get rid of the poison, then an endeavour must be made to neutralise what remains, and it is here that the question of antidotes comes in; antidotes being anything acting in the opposite direction to the effects of the poison. Thirdly, at all risks the patient must be kept alive. It must always be remembered that time is of the utmost importance in all cases of poisoning, as every lost moment renders treatment more difficult and recovery more remote.

In regard to poisons, all text-books begin with acids and alkalies, but what these terms convey to the young nurse or probationer is not very distinct. Our classification of poisons shall be into those which are found within the institutions or house—favoured possibly by an oversight on the part of the nurse, and of those which may be found and taken out of doors. Of the former the so-called acids may be considered first. These are mainly corrosive poisons, and in their action they burn and destroy the tissues, the commonest of them being—(*a*) Oil of vitriol or sulphuric acid, used in charging the accumulators of electrical plant, in the manufacture of soda-water, and for domestic purposes. (*b*) Aquafortis, red nitre or nitric acid, a fuming brown liquid kept for treating warts or other caustic effects. (*c*) Spirits of salts, muriatic or hydrochloric acid, used for tinsmiths' purposes and brass polishing. (*d*) Salts of lemon, bonnet acid, acid of sugar or oxalic acid, a crystalised substance like Epsom salts used as an ingredient in polishing pastes, for removing ink stains, cleaning hats, and by grooms and bookbinders for cleaning leather articles. (*e*) Carbolic acid, used for disinfecting purposes, for stings and bites or for curing toothache. All the above are corrosives; some, such as aquafortis, leave brown stains on the lips, mouth, and skin, some also (like vitriol) a black stain, whereas others, such as carbolic acid, spirits of salts, and oxalic acid, leave a white stain.

For corrosives it is best not to administer an emetic, because the strain and efforts at vomiting may cause a rupture of the œsophagus or the stomach; but antidotes, such as baking soda or ordinary washing soda, lime, chalk, whiting, or whitewash scraped from the ceiling and mixed with water, are to be administered, followed by olive oil, or castor oil and milk, or milk gruel. Epsom salts and the white of an egg beaten up with water are the best remedies for poisoning by white precipitate or corrosive sublimate (of mercury), as also for poisoning by saltpetre.

In all cases where collapse is a marked feature, warmth to the feet and warm water with brandy must be administered so as to keep the patient alive. Death from prussic acid is so rapid that no antidote is of any avail as a remedy, but artificial respiration must be employed so long as there is any hope of life. As to alkalies—such as ammonia, hartshorn, caustic potash, or "pearl ash," caustic soda—used as rat poison—their general treatment is to give vinegar freely diluted and alternately with olive oil.

Alcoholic poisoning is too well known to need special attention, for this, together with other spirits, such as turpentine and laudanum, or opium, an emetic is necessary, followed by strong coffee and compulsory exercise, such as walking about between two nurses, flipping with wet towels, and the use of the electric battery. For antimony, used for furniture polish; or horses' medicine; or for poisoning by arsenic, as in fly-papers or vermin killers; for zinc powders, used as hair dyes, an emetic is administered to promote vomiting, strong green tea, white of egg with milk and water, oil and lime water, are also given to neutralise the effects of these poisons.

Sleeping draughts of chloral or chlorodyne in excess may also cause poisoning. The treatment is to give an emetic, such as mustard and water, keep the body warm, give strong coffee, and arouse the patient so as to prevent sleep getting the mastery. After chloral, but not after opium, allow the patient to be in bed and at rest. No exercise is necessary nor is it desirable after an over-dose of chloral, its action being to lower the pulse and weaken the heart's power. For poisoning by the salts of copper an emetic is administered, and fomentations applied for abdominal pain. For lead, an emetic with Epsom salts. For caustic (lunar caustic) or nitrate of silver give salt freely dissolved in water. For mussel or shell fish, paraffin oil or petroleum poisoning, give an emetic and take means to prevent collapse. For phosphorus—the paste often used for rat poisoning—which is sometimes taken with the intention of destroying life, give an emetic of sulphate of zinc; in this case do not give oil, as it dissolves the phosphorus and may thus assist its absorption. For irritant poisons, the administration of an emetic, followed by the free use of milk, are as much as the nurse can do. The question of antidotes is generally a matter for the medical officer.

It may be that poisoning may occur accidentally when the patient is out of doors, taking exercise or sitting about in the grounds, some herbaceous or garden plants, the leaves of trees or seeds, being swallowed thoughtlessly or with intent, and they yield the second group of poisons for our consideration. Among these may be mentioned the fungi or poisonous mushrooms; aconite, known as "monk's-hood" or "blue rocket," the root of which has been on more than one occasion mistaken for horse-radish. Belladonna, known as "deadly nightshade,"—the fruit being a blackish, purple berry—has also been taken accidentally. Bryony, digitalis or foxglove, hemlock or "fool's parsley," privet, holly, laburnum pods and seeds, the flowers of laburnum trees called "golden chain" or "golden rain," lords and ladies, cuckoo pint, and yew leaves—may all be taken in poisonous quantities and must be treated by an immediate emetic, followed by castor oil and milk, or coffee if there be drowsiness, and stimulants if there be any loss of strength or a tendency to collapse.

In poisoning by coal-gas, which may occur from an escape into the sleeping apartment, artificial respiration must be at once resorted to in the open air, smelling salts applied to the nostrils to excite breathing, movements and stimulants given—the whole time keeping the body warm. In the case of stings and poisonous bites, if possible first take out the sting, then wash the wound, rub with Scrubb's ammonia, or some essential oil such as lavender or eucalyptus. Tie a ligature on the "heart side" of the sting of poisonous flies, snakes, or vipers, and "keep the patient alive" by careful wrapping up of the body in warm blankets, or by applying hot-water bottles to the extremities; also, for collapse, administer stimulants with caution and deliberation.

*(To be continued.)*

### The Training of Mental Nurses on Hospital Lines in Theory and in Practice.

Ten years ago Dr. W. F. Menzies, who was at that time senior assistant medical officer at Rainhill Asylum, read a paper before the Medico-Psychological Association advocating the improvement of the status of the nursing staff in asylums. In this paper Dr. Menzies contended that, given that some change in the conditions of labour is necessary, it appeared to him that three ways were available. He affirmed that the field of attraction for candidates might be enlarged by making it worth while for individuals of a class where the struggle for existence is keener to enter; that the engagement of persons of a class willing to enter domestic service, for whom the demand exceeds the supply, should cease; and that inducement might be offered to better-class girls to stay for at least three years' training. In order to raise the status of the asylum nurse and enable the institutions to improve the quality of the staff, he maintained that certain reforms were indispensable.

### The Age of the Probationers.

To begin with, he said, the minimum age of probationers commencing training must be raised, and in support of this plea he pointed out that the wear and tear of ward work reacts with greater force on immature girls, and that the younger nurses in asylums were most often laid up with trifling ailments. He insisted that if physical maturity was requisite for general hospitals, it was much more indispensable for asylums, while the mental development which years and experience alone bring was necessary in the latter but not in the former. In indicating his preference for a minimum of 25 years, he admitted that there were arguments in favour of 23; but in objecting to fractious delusional or fussy senile cases being handed over to the tender mercies of girls of 19 to 21, he remarked that the youth and frivolity of the nurses, and their want of tact and judgment, were a not infrequent cause of complaint on the part of patients when quarrels arose.

### The Hours of Duty.

Dr. Menzies went on to urge the necessity of shorter duty hours and longer daily and annual leave. He suggested an amended daily time-table with the day beginning at 6.30 instead of 6, and ending at 11 instead of 10, the daily time off so arranged that the total working hours per week would be 72¼. With this allowance of time off, he considered that nurses might with justice be asked to attend occasional lectures, patients' amusements, rehearsals, and choir practices in their own time. Annual leave, he recommended, should be increased from 14 to 21 days in the year, charge attendants and nurses being allowed a month. In order to more effectively carry out these proposals, which would, generally speaking, involve about an addition of a third to the staff for day duty, he advised the employment of wardmaids, not because ladies would refuse any menial work, but because it was false economy to employ them to clean wards, single rooms and passages, when the work could be as well done by untrained maids.

### The Improvement of the Dietary.

While conceding that the quality of the food was satisfactory and the quantity ample, Dr. Menzies averred that diet-sheets lacked variety, that dishes were often spoiled by poor cooking, and that they were not nicely set upon the table. He held that the dietary should attain a public-school standard, or probably, allowing for the proverbial difference of appetite, aim a little higher, and said he was convinced that in a French institution, from such excellent material as is supplied in English asylums, a very much better result could be obtained at two-thirds of the money, and that the extra expenses of a larger kitchen staff would, in a few years, be more than repaid.

### Homes Separate from the Wards.

Even 10 years ago Dr. Menzies said that the provision of a separate block for both nurses and attendants was an axiom with superintendents; but in emphasising the importance of homes he observed that rooms should be furnished in them for at least 100 nurses in the case of a large asylum, so that none should be compelled to sleep in the wards, but that each might be in a position when off duty to entirely dissociate herself from insane environment. With regard to night nurses, of whom he said there should be two for every 100 patients, he saw no reason why these in quiet wards should necessarily sit in the patients' dormitories, and they should be able to give help at any time in the acute wards, by means of open telephones with microphone transmitters and receivers communicating with the office of the night superintendent. With regard to recreation he contended that one of the most important spheres of duty of assistant matrons should be to look after and encourage the social life of the nurses, and readings, concerts, sewing classes, and small dances could be held almost nightly in the home. In the male block the assistant medical officers should preside at various symposia.

### A Complete Course of Training.

Dr. Menzies then dwelt upon the importance of more complete instruction in hospital nursing and the management of insane persons. He acknowledged the improvement in nursing which had taken place since the training scheme of the Medico-Psychological Association had been founded in 1890, but suggested that a further development might be sought in affiliation for teaching purposes to general hospitals, so that probationers, in their second year, could go there for some months to obtain that wider grasp of medical and surgical nursing which no asylum could supply. In the third year they would prove very valuable to the asylum, and could thereafter receive the certificate. He pointed out that private nursing of mental cases was a lucrative calling, and expressed his belief that if some hospital training went with mental experience the original outlay would be soon repaid. Finally he went into the question of cost, which he estimated might mean an addition of 1s. 3d. in the maintenance rate, and that even then the average cost of the insane in county and borough asylums would be 10s. 5d. per week as against 16s. 11d. in the United States.

### Practice at Cheddleton.

In 1899 Dr. Menzies was appointed superintendent of the Staffordshire County Aslum at Cheddleton, with 618 beds, and he considered that the opening of a new institution afforded him a favourable chance for carrying his ideas into operation. Therefore, from the first, the hours and duties at Cheddleton were modelled to suit the employment of ladies. To every applicant for a post a circular setting forth the duties, scale of pay, and condition of engagement is forwarded.

The preface is as follows:—"The duties of nurses consist, in the fullest sense of the word, in attending to and caring for the patients under their charge, and by every means in their power endeavouring to promote their welfare and recovery. The sick require to be nursed, the helpless washed, dressed and fed, the excited pacified and restrained, the depressed stimulated and encouraged. Walks in the grounds and country, tennis and croquet, concerts, dances and theatricals, and choral service on Sundays, are severally useful in taking the patients' thoughts out of themselves, and thus directly benefiting them. Nurses, therefore, who possess qualifications suitable for the above are, provided they possess the necessary tact and intelligence, of special value to the institution."

### The Three Years' Training.

The circular says that in order to equip ladies for taking up the profession of private nurse in mental diseases, a complete three years' course of training is offered to probationers in connection with the nursing certificate of the Medico-Psychological Association of Great Britain and Ireland, comprising:—1st year, Anatomy and physiology, ward duties, general care of patients, the elements of sick nursing. 2nd year, The outlines of medical and surgical ailments, with their symptoms and treatment, more advanced nursing of ordinary medical and surgical cases, fractures, operations. 3rd year, Anatomy of the nervous system, psychology, pathology, and treatment of nervous diseases, special sick nursing of nervous disorders, sick cookery, moral and intellectual mind training, massage, and electro-therapeutics.

This course of training is compulsory upon all probationers, and none are permitted to enter for the nursing certificate who have not fulfilled it. Those who cannot satisfy the medical officers of their diligence and attention during a junior course are not allowed to enter a senior. The qualifying examination is not compulsory, but no one can be recommended for a senior post, either at Cheddleton or elsewhere, who has not passed it.

### Salaries and Duties.

The salary of probationers during their three years' training is £20, £22, and £24 per annum respectively, with board, lodging, and washing. When probationers have passed their qualifying examinations, if they desire to remain in the service of the institution, and providing vacancies exist, they are eligible for appointment as nurses at £26 a year, or more according to ability. Staff sisters receive at least £4 per annum in addition. Increases of salary to nurses and sisters are regulated by good conduct and ability, until a maximum of £50 is reached. As a matter of fact, sisters begin at £33, with £36 the next year. Probationers are provided with uniform dresses, aprons, collars, cuffs, caps, bonnets, and cloaks, after they have signed an agreement for three years of training.

The daily time table, which is liable to be altered at any time without notice, is:—7 A.M., nurses' breakfast (in mess-room); 7.30 A.M., commence duty; 12 noon to 12.40 P.M., first nurses' dinner (in mess-room); 1.10 to 1.50 P.M., second nurses' dinner (in mess-room); 4 P.M., afternoon tea (in wards); 8 P.M, go off duty, nurses' supper (in mess-room); 11 P.M, lights out. Anyone desiring lunch at 10 A.M. may have it on application to the matron.

Probationers and nurses are allowed out in the evening by leave of the matron, and late leave until midnight or later can be occasionally obtained for the purpose of attending concerts, theatricals, or dances outside the building. Probationers and nurses are on evening duty about once in eight or nine days.

As far as the exigencies of the institution and the strength of the staff permit, probationers and nurses are allowed off duty from one to two hours daily. Probationers have two and a half days and nurses three days off every month. The annual holiday for probationers is fourteen days, for nurses twenty-one days, and for sisters a month when they can be spared.

The pension scheme in force is founded on one-fortieth of salary and emoluments for each year of service, up to a total of two-thirds as allowed by the Lunacy Act.

### The Set-back to the Scheme.

It never occurred to Dr. Menzies that there would be a dearth of applicants, and yet he states at the end of five years that this has been the only set-back to the scheme. There are now about 30 of the better class of nurses on duty, but he requires an average of 40. He attributes the deficiency in the supply to the several fallacies which, he thinks, pervade the public mind. One is that the work is very laborious. Dr. Menzies himself regards it as a good deal easier than the work at the general hospitals. Not only is there a ward-maid in each ward at Cheddleton, but practically all the hard menial work is done by patients under her direction, so that the nurses have nothing to do except to attend to their patients. He thinks that the hours, which are a good deal modified from those he proposed in 1894, compare well with the hospital average, while in addition to this a great deal of the time is occupied with supervision rather than with the manual labour of attending to nursing operations. Another fallacy he mentions is that mental work is of no use afterwards. His answer to this is that he can ask in the Cheddleton district 3½ guineas per week for acute cases, and could send out six times the number of nurses if he had them; and he adds that there is an enormous field for the highly-trained mental nurse of superior education. With only about 100 sick beds, and few major surgical cases, it is not pretended that the necessary experience for abdominal cases is given at the Cheddleton Asylum, but the authorities undertake to provide the best mental training obtainable in three years, together with enough general work to competently nurse any physical complication in a mental case, whether surgical, medical, obstetrical, or acute specific.

### The Standard of Knowledge.

As to the standard of knowledge sought to be instilled, the medical superintendent uses Murché's "Elementary Physiology" as a text-book for the first year, with any of the usual books on nursing for that of the second, supplemented by whatever the assistant medical officers think needful. The matron, who was trained at the Liverpool Royal Infirmary, holds systematic classes in practical nursing.

A short time back Dr. Menzies approached the Liverpool Royal Infirmary with the view of seeking affiliation to it for teaching purposes, but was met with a point-blank refusal. In the third year he enters very fully into the anatomy and physiology of the brain and nerves, and recommends Mercier's "Insanity" as a text-book. Each year about 50 lectures are given, with about 40 demonstrations in nursing by the matron and assistant medical officers. The lady superintendent gives demonstrations in sick cookery. Each nurse has a separate bedroom, and there are the usual recreation-rooms in the small block used by nurses attached to the building. Though there is at present no nurses' home apart from the asylum, one is included in the plans of extension, and will be built as soon as funds are available. The extension referred to will raise the number of beds to 1,000. When Dr. Menzies formulated his scheme he did not anticipate that it would be necessary to pay probationers, and the cost as originally estimated has therefore been exceeded at Cheddleton. As an appendage to the nursing school there is a small school of cookery, under the lady superintendent and a qualified assistant. Pupils are instructed in domestic and institution cookery, catering, housekeeping, and, to a certain extent, in fancy cookery, for one year. They pay no premium, but do not receive a salary. They are by that time quite capable of taking a post as lady cook in a small institution or large middle-class country house, although it is found that they still require, as a rule, additional practice in high-class cookery before they can be described as thoroughly efficient as teachers or chefs.

### Christmas in the Hospitals.

BY OUR OWN COMMISSIONERS.

*Concluded from page 195.*

### St. George's Hospital.

Christmas dinners were as usual provided for all those patients who were well enough to enjoy them at St. George's Hospital, and were partaken of on Christmas Day, when the wards were decorated with evergreens, fairy lamps, etc. The Christmas tea for the patients took place on the 28th, when tea and coffee, fruit, cakes and other suitable delicacies were provided, and the lady visitors and various friends distributed gifts, both useful and ornamental. Additional pleasure was caused by the fact that Princess Victoria of Schleswig-Holstein was present on the occasion and took part in the distribution. Among the toys for the children were some sent by the proprietors of *Truth*, and there were trees in the wards where there were children. On the following day there was a conjuring and musical entertainment in the board room, at which all who were able to walk or to be carried down on stretchers were present. The number was somewhat smaller than usual, owing to the fact that a good many patients had just left for the convalescent home; but the entertainment was heartily enjoyed by all who were present.

### King's College Hospital.

Christmas was ushered in at King's College Hospital by the singing of carols in the central hall at midnight, and later the patients awoke to find presents on their lockers. There was an early morning service on Christmas Day, and at the five o'clock service carols were again sung. The Christmas dinners were disposed of on Monday by the patients; Tuesday being the day devoted to the sisters and nurses. On Wednesday the annual concert and variety entertainment took place. A musical programme was provided by the nurses and students, who visited each ward in turn, beginning at 3 30. Then came a band of pierrots—a coloured edition of the type, but adorned with the familiar sugar-loaf hat and indispensable balls—who gave admirable renderings of such part-songs as "The Baby on the Shore" and "Down among the Dead Men." Great amusement was occasioned by the topical allusion to the projected removal of the Hospital to a new site, in the lines :—

"Up the hospital stairs you do clamber well,
But soon we are going to shift;
And if you will come down to Camberwell,
You shall go up and down in the lift."

A very amusing performance of Mrs. Jarley's waxworks concluded the round of entertainments, the old lady appearing in grey corkscrew curls and large spectacles. She was assisted by Peter and John, and announced by a pair of decayed sandwich men. The figures included "You Dirty Boy," "Sandow," "Beware of Imitations," "Where are you going to, my Pretty Maid?" and the strong baby fed on patent food, in muslin frock and pink ribbons, who succeeded in raising the fainting policeman from the floor and running with him out of the ward, a feat greatly applauded by the delighted spectators. Part of the performance was repeated on Thursday, when the children in Panta Ralli ward had their presents from a magnificent tree, lighted for the first time by electricity. This ward was very prettily decorated by window boxes planted with paper daffodils, as well as by real arum lilies and chrysanthemums. Twining ward was festooned with pink roses—the work of the patients—and there was a trophy of flags in the central hall.

### St. Mary's Hospital.

Every patient at St. Mary's Hospital received a useful present on Christmas morning. The children had giant stockings hanging in their cots filled with all sorts of toys. Great was the glee as the little ones kept on discovering fresh treasures. "Tum here and 'ook what I've dot," was the constant cry. The male patients were allowed to smoke; even in the casualty room pipes and tobacco were provided. Christmas Day was kept as a Sunday, but the dinner was real Christmas fare. The nurses' dinner consisted of roast turkey, plum pudding, and a most liberal dessert; this was presented by a generous friend. Boxing Day was the gala day; entertainments were given in every ward from 4 to 8 P.M. Music of all kinds was going on, and a stray gramophone found its way into a ward and discoursed for an hour. Crawshay, the children's surgical ward, looked lovely—yellow and white were the reigning colours. Paper flowers—home-made—were the rage everywhere, but those in Crawshay were the most highly finished. The children wore pale-blue jackets. One little boy was sobbing bitterly after the visiting hour was over. "Never mind, Tommy," said the little girl in the next cot; "when your leg gets better you'll be able to go home; then you'll see your mother all day long, and you'll go back to school, and you won't be washed three times a day—think of that." "Oh," sobbed Tommy, "I don't want to go to school, and I don't want to have a dirty face no more." The annual Christmas Tree was held in the board room of the hospital at 4 p.m. on Tuesday. All the children who could be moved were brought down, and several were invited who had been in-patients in previous years.

### Hospital for Sick Children.

"Tree Day" at the Great Ormond Street Hospital for Sick Children passed off very successfully on Thursday last

### CHRISTMAS IN THE HOSPITALS—*Continued.*

week. The building was crowded with visitors, and many Christmas gifts had been received by the authorities, including toys from the Princess of Wales, *Truth* toy fund, and a large number "to the memory of Corney Grain." A handsome gift of fruit from the Dominica Agricultural Society was also received. This fruit was offered to the King for his acceptance for the London hospitals, and, at his suggestion, was sent to the Hospital for Sick Children. Amongst the fruit were some 11 crates of bananas, many boxes of limes, navel oranges, grape fruit, citrons, and apples. The last were grown by Lord Aberdeen on his ranche at Vernon, British Columbia, and exhibited by the Agent-General of British Columbia at the recent Royal Horticultural Show. There was no general entertainment, but each ward had its own beautiful Christmas tree, shimmering with tinsel, lit either by small candles or tiny electric lamps, and literally weeping with toys. "Clarence" Ward looked very pretty with its fairy lamps and flowers and paper gas shades. The "Corney Grain" Cot, now occupied by a tiny baby, is in this ward. "Louise" looked very bright and charming, while "Helena" was most fairy-like, its pretty glass ward tables affording excellent scope for dainty table decorations and reflecting the daffodils and greenery which were used for those decorations. "Victoria" had plenty of fairy lamps and very pretty flowers; while the decorations in "Alice" were most original, including an effective Chinese ornamentation on the flat top of the central fire stove.

### The Cancer Hospital.

A very happy Christmas was spent at the Cancer Hospital, Fulham Road. On Christmas morning the sisters, nurses, and maids, each carrying her own lighted candle, met outside one of the wards at 5·45 A.M., and a tour of the hospital was made while carols were sung to the patients, the entire round occupying about an hour. Little dainties were added to the patients' breakfast, and at dinner every ward had its turkey and plum pudding. A sumptuous tea was provided for the nurses in the new home at 5 P.M., after which there was a service in the chapel, when carols were again sung. On Monday festive teas were in full swing from 4 P.M. to 6.30, while friends of the hospital contributed a musical programme. Each patient was allowed to invite two friends to tea, and there was a magic lantern in Ellis Ward. The decorations were exceptionally dainty, and included branches of mistletoe, water lilies, red japonica, hawthorn, and daffodils, the paper flowers being the work of the patients, with the aid of twigs cut from the garden. Presents were provided for sisters, nurses, patients and maids.

### Brompton Hospital for Consumption.

The Christmas entertainment at Brompton Hospital for Consumption took place on Thursday amidst great rejoicing. A beautiful Christmas tree was presented by Miss Heddy. The tree, which was laden with presents for all the patients, was lighted by electric lamps. Father Christmas (Dr. Inman, one of the house physicians), Dr. Paterson (resident medical officer), Miss Lloyd-Still (the lady superintendent), and several nurses distributed the gifts. Besides the tree there was also an excellent cinematograph, which gave much pleasure. There were many visitors who shared the enjoyment of the patients.

### East London Hospital for Children.

The Christmas entertainment for the patients of the East London Hospital for Children at Shadwell took place on Friday. The building was profusely decorated with Chinese lanterns, flowers, and evergreens, and a large number of visitors were present at the marionette entertainment in the out-patients' department. The wards were practically deserted, most of the children being able to come to the entertainment, though a number of them were obliged to lie on mattresses on the floor. After the marionette show the two large Christmas trees standing at the far end of the room were dismantled, and Father Christmas, assisted by several nurses, gave presents to the children, whose excitement and pleasure were unbounded. During the entire afternoon the band of the Royal Horse Guards played a very nice selection of music. A decided attraction was a beautiful St. Bernard dog belonging to the nurse in charge of the electrical department, who, by means of a small box strapped on to his back, and graciously allowing people to pat him, collected money for the institution. Outside the hospital gates a group of ragged little children eyed wistfully some visitors who were leaving, and one as spokeswoman, said: "Ain't it nice just in there? I wish I was in the 'orspital!"

### Mount Vernon Hospital for Consumption.

At the Mount Vernon Hospital for Consumption, Christmas Day was kept as an ordinary Sunday, but on Bank Holiday the patients all dined together in the large dining-hall, which was beautifully decorated. Amongst the visitors were Mrs. Brook Deedes, Mr. Charles Johnston, Mr. Henry Stedall, and Dr. Williams, the House Physician. During the afternoon an excellent concert was given by Mr. Charles Johnston and friends. At tea-time four of the nurses, dressed respectively as Father Christmas, Dame Christmas, the Old Year, and the New Year, gave presents to everyone. Later in the day the patients had a concert of their own.

### The Lying-in Hospital.

Everything possible was done to gladden the hearts of the poor East End mothers who found themselves inmates of the City Road Lying-in Hospital on Christmas Day, and complete success attended the untiring efforts of the matron and nursing staff to make gay and bright the wards of this most excellent institution. Early and late, the day before the neighbouring church bells ushered in the great festival of the Christian year, willing hands worked deftly with holly and evergreen, twisting the boughs and twining the leaves into every kind of device emblematic of the occasion. Others planned and made ready the old familiar mottoes which at this season stir the soul with thoughts of charity for all mankind. At length all was in order and the finishing touch put to the numerous decorations which greeted the astonished and delighted patients on Christmas morning. Sweetly pretty were the tiny cots, each with its wreath of holly, toning in harmony with the red and white drapery; while peeping from beneath the coverlet was the pink chubby little face of baby. Very striking also was the scene presented by the nurses themselves, who, bearing candles of various hues, held fast in coloured holders, walked about the wards in slow procession singing carols and songs of praise. The convalescents were allowed a very liberal repast, and for those unable to sit up dainty meals were prepared by the nurses in charge. On Monday afternoon a concert was arranged for the patients. Next came the turn of the nurses, who passed a very happy evening together.

### Hampstead General Hospital.

The Christmas festivities at Hampstead General Hospital were spread over several days. On Christmas morning, Miss Gregory, the matron, and the nursing staff, gave presents to all the patients. In the afternoon Miss Frankau brought tobacco and pipes to the men, sweets to the women, and toys to the children. On Bank holiday evening the staff dined together, and on Wednesday afternoon each patient had two friends to tea. After tea there was an entertainment at which the nurses sang a carol, Miss Sanderson and Mr. Kreuser played piano and violin duets, Mr. H. Shearsmith and Mr. J. S. Nott-Barnes gave banjo duets, and Mr. Percy Castello contributed a number of humorous musical sketches.

ST. MARYLEBONE INFIRMARY.

Thursday in Christmas week was the day chosen for the annual inspection of St. Marylebone Infirmary, when tea was provided for visitors, who were invited to go through the wards. Chinese lanterns, flags, and evergreens had been effectively used in the long corridors, and down each opening a pleasant vista of lights and colour met the eye. Tea parties, either at central tables or at the bedside, were in full swing, and there was plenty of good cheer for all. A rich profusion of mottoes and emblematic designs clustered on every wall, and seemed to sprout from every spare corner. Outside "G 3" (men's ward) sat Darby and Joan, fat and complacent, congratulating one another on the fact that another Christmas was being spent together. Inside, a Japanese turn of mind had suggested fans, screens, and chrysanthemums, while under a large umbrella there stood a life-size Japanese soldier in a complete suit of armour. "The Macedonians" gathered round a scanty camp-fire in another ward. One of the galleries, where the open-air treatment is being carried out, blossomed into festoons of greenery, flags, and lanterns for the occasion the effect, under canvas, being somewhat nautical. During the evening the nurses and staff visited the wards and sang carols, to the great delight of the patients.

SALOP INFIRMARY.

Christmas rejoicings commenced at the Salop Infirmary at 6 A.M. on Christmas Day, when the nurses sang carols through the corridors. The wards were beautifully decorated by the doctors, sisters, and nurses. Miss Pegg (matron), accompanied by the house surgeon, the latter dressed as Father Christmas, distributed useful gifts to each patient during the morning. Father Christmas caused much merriment amongst the patients. On Monday the patients had their Christmas dinner—beef, chicken, pudding, and fruits. They all spent a very happy day. Music was amply provided in all the wards, songs being sung by the doctors and nurses. On Tuesday afternoon there was a large gathering for the Christmas tree, which was loaded with presents for everyone. The tree was placed in the surgery, which was turned into an impromptu theatre for the week. Marionettes and an amusing costume sketch by the nurses afforded much pleasure and amusement to all. During the afternoon the visitors partook of tea, which was provided by the matron in the board-room. On Wednesday the day nurses gave Roeckel's operetta "The Gitana." The effective dresses and scenes of Spanish court and gipsy life were greatly admired and enjoyed by the patients and guests. The night nurses contributed two plays, entitled "Too Many Cooks" and "A Nice Quiet Chat." Both of these provoked much merriment.

ROYAL ISLE OF WIGHT COUNTY HOSPITAL.

The wards of the Royal Isle of Wight County Hospital were tastefully decorated with evergreens, lanterns, and fairy lights. On Christmas Eve the nurses sang carols in the ward corridors. Christmas Day was celebrated on Monday, most of the patients being able to partake of turkey, plum-pudding, and jellies. Dinner was followed by a visit from Mr. and Mrs. Bill Bailey to distribute presents to the patients. In the evening a coon concert was given by the nursing staff. The cake walk was danced by two nurses. On Wednesday the Christmas tree took place in the children's ward, the adult patients receiving clothing and the children toys. In the evening two "plays," entitled "A Perfect Cure" and "Two Naughty Old Maids," were performed.

### Farewell of the Lady Superintendent of the West London Hospital.

BY AN OLD WEST LONDON NURSE.

ON the last evening of 1904 Miss Irene Hardy gave a farewell party to the staff of the West London Hospital, past and present. The guests assembled at 7 P.M., and the spacious out-patients' hall had been converted into a concert-room, a platform, decorated with flowers and palms, being erected at one side and seats conveniently arranged all round.

An excellent programme had been prepared, divided into two parts to enable both night and day staff to enjoy some of the festivities. There were songs, humorous and otherwise, musical sketches, whistling solos, recitations, and last but not least, most amusing and wonderful performances by Mr. C. Bertram, the prestidigateur. At the end of the first part Dr. Donald Hood, the senior physician of the hospital, mounted the platform and in a few well-chosen words explained that the gathering, though so enjoyable, was not altogether unmixed with sadness, as it was the occasion of their farewell to Miss Irene Hardy, with whom some of them had been connected for 25 years and all present for some time, and who, by her unfailing kindness and the good work she had done for the hospital, had endeared herself to them all. He then inquired for Miss Hardy, who was escorted to the platform by Mr. Gilbert, the secretary, and asked her to accept, as a mark of affection and esteem from the medical staff, past and present, a handsome solid silver tray and a gold curb bracelet.

Miss Hardy thanked the donors of this handsome gift, saying that she should use the tray every day and wear the bracelet always. Mr. Lloyd, the anæsthetist, added a few remarks of appreciation to those already made by Dr. Donald Hood. Mr. Gilbert then, on behalf of the nursing staff, past and present, the office, pathologists, dispensers, servants and porters, presented her with a fitted folding writing-table, bearing a suitably inscribed silver plate, a travelling rug, a purse of gold and a Russian leather bound volume containing the names of all who had subscribed, autographs where possible. Miss Hardy, who was deeply moved, as indeed were all present, said that she valued the affection and esteem of her kind friends even more than their beautiful gifts, and spoke of the happiness it had been to her to work there for so many years.

In addition to the presentations made on December 31st, Miss Hardy had already received from the Hospital Committee a silver-fitted dressing-bag, a travelling clock, and an illuminated testimonial. At the top is a water-colour sketch of the hospital, and on either side a scroll of conventional leaf design, with the following statements in a ribbon on the sides:—

"Admitted as Nurse Probationer, 29th September, 1879 Appointed Hon. Lady Superintendent, 10th January, 1881."

"Appointed Lady Superintendent on Paid Staff 1st July, 1890. Appointed Matron, 15th March, 1897."

The centre is taken up with the following words:—

"Presented to Miss Irene Hardy upon her Resignation as Matron of the West London Hospital by the Board of Management as an expression of its very high appreciation of valued services rendered by her during the 25 years that she has been on the staff. The record of her association with the hospital is in itself a proof of the high esteem with which the management of the hospital has always regarded her, and in parting from her the Board sincerely hopes that she may be spared for many years to enjoy the rest so honourably earned by her faithful devotion to the noble work of tending the suffering poor."

It was a striking testimony to the popularity of Miss Irene Hardy amongst the medical staff that, with the exception of three, who sent telegrams of regret, the whole of the former and present physicians, surgeons, and anæsthetists were present at the presentation.

### Everybody's Opinion.

### REFORMS NEEDED AT CHELTENHAM HOSPITAL.

The Chairman of the Grimsby and District Hospital writes: In The Hospital of December 24th I read some notes on the Cheltenham General Hospital, in which the following remarks appear: "The nurses both in the hospital and in the home for private nurses, sleep in cubicles. This, although the cubicles are comfortably furnished, is a serious drawback." Why? The reason I ask is that we are now building a new home for the nurses and it was our intention to provide cubicles as being more healthy and giving a free ventilation. We cannot afford to make a mistake and it is not too late to alter. Will you kindly give us the benefit of your opinion?

### CLASS LEGISLATION.

"Inquirer" writes: If "One who cannot be Registered" will procure the "Minutes of Evidence taken before the Select Committee on Registration of Nurses," she will see for herself who would or who would not be eligible for registration, and for what class of women the promoters of the Bills wish to secure the better-paying patients if they can. I have not my copy of the evidence with me, but if I remember correctly, the term of grace suggested was two years for the earlier-trained nurses. If those nurses who are agitating for registration and are not eligible themselves quite understand what they are about they must be noble, unselfish women, and I hope the more fortunate ones will appreciate their sacrifice; but I have my doubts. Still I am sure that there is no need to worry. Registration is not wanted by hundreds of doctors, who are the nurses' friends and know by experience what they can do, and sometimes make comparisons not favourable to nurses with a three years' training in a large hospital. It depends a great deal upon the individual. I was amused by the opinion of a patient's relative who was discussing the nurses who attended his relation: 'Yes, Miss —— was very ladylike and smart; wore stiff, rustling, starched dresses, large cuffs, etc. was clever, could talk and diagnose a case like a doctor, but she could not nurse.' She will be on the State Register, if there is one. It is nonsense to argue that registration will prevent scandals in the nursing world; nurses are human and, among the large number practising, we cannot expect all to be perfect. Certainly a three years' certificate and having their names on a State Register will not make them so."

### MEDICAL MISSION WORK OF THE S.P.G.

Mrs. Bickersteth writes:—The Committee of Women's Work of the Society for the Propagation of the Gospel have desired me, as one of their hon. secretaries for candidates, to ask if you will kindly allow space for a few words about the medical mission work to which kindly reference was made in your columns on December 25th, in connection with Mrs. Creighton's recent speech at Exeter Hall. Probably many of your readers are aware of the suffering and misery endured by our sisters in most non-Christian lands, notably in India, in times of illness when left to the "tender" mercies and ignorant nursing of their own people. I have recently spent a few weeks in India, and I think I can never forget the glimpses I had of women rescued from this suffering and tenderly cared for in our Mission hospitals. Of these hospitals there are all too few at present, and those we have are but slenderly staffed in proportion to the work with which they have to cope. We do earnestly ask for funds to extend this side of our work, in which those who are engaged seem to be treading in the very footsteps of the Master, and for volunteers to carry it on efficiently. By medical missionaries of the S.P.G. we understand doctors and nurses, earnest and devout members of the Church of England, who, having received a full and complete professional training, are prepared to go out and exercise their profession in the name and for the sake of Christ, and by their lives of loving service to commend to their non-Christian sisters the faith which they represent. We do not as a rule expect or desire our medical workers to give direct evangelistic teaching, but they must be in full sympathy with this side of the work, and must themselves be missionaries in deed and life if not in word. Their opportunities for influence, whether among their patients or with the young Christian girls who come to them for training, are practically unbounded. Are there not some among your readers at the opening of this new year who will claim the great privilege offered them of becoming light-bearers to those who in a very real sense sit in darkness and in the shadow of death? Are there not others who, unable to go themselves, are yet desirous of showing sympathy and practical interest in this branch of the Church's work? We know of some hospitals where friendly talks about missions are welcomed, and where as an outcome of such talks much practical help is given by the staff. Invitations to pay such visits would find a cordial response at the S.P.G. office. All inquiries, etc., should be addressed to the Secretary, Committee of Women's Work, S.P.G. House, 19 Delahay Street, Westminster.

### Appointments.

Stanley Hospital, Liverpool.—Miss Mary Davies has been appointed assistant matron. She was trained at Guy's Hospital, London, and has since been night superintendent at the Royal Southern Hospital, Belfast.

### "The Hospital" Calendar.

Last year so many thousands of nurses showed appreciation of our little calendar and note-book that we have this year prepared a neat little case into which it can be secured, and hung by an attachment to the chatelaine if desired. "The Hospital" Calendar is in appearance a miniature representation of The Hospital Journal. It contains besides useful information sufficient space for notes on every day of the week. The calendars can be obtained, price 2d., post free, and with covers, price 3½d., from the Manager, 28 Southampton Street, Strand.

### Our Christmas Distribution.

The Matron of the London Hospital writes: "I desire to thank you and the readers of The Hospital for the clothes which have been sent by you and by them for the use of our patients. We are exceedingly grateful to you for your kind thought, and much regret that, owing to the pressure of Christmas work, we have not sent you an earlier acknowledgment. We are always very grateful to friends when they help us to provide clothes for our patients, as they are often so sadly in need of them."

### Echoes from the Outside World.

### Movements of Royalty.

After spending Christmas and New Year's Day at Sandringham the King and Queen, accompanied by Princess Victoria, left Norfolk on Monday evening on a visit to the Duke and Duchess of Devonshire. Their Majesties arrived at Rowsley Station, where the electric light has been specially installed, at five o'clock, a charming effect being produced by hundreds of coloured electric fairy lamps hidden among the shrubs and foliage which cover a sloping bank just outside the station. The Royal party were met by the Duke of Devonshire, but a guard of honour at the station was dispensed with. On the arrival of their Majesties at the great gates at Chatsworth, the workmen employed on the estate assembled, and formed a procession of torch-bearers. The other guests of the Duke and Duchess include the Prime Minister. The King being desirous of keeping in touch with public business, a special wire has been established from Bakewell to Chatsworth House so that he is in direct communication with London. On Tuesday the King enjoyed some pheasant and wild duck-shooting, at Bunker's Hill, one of the highest points in the neighbourhood, and rode back to Chatsworth House on his pony. The Queen spent a portion of Tuesday afternoon watching the players on the golf links, who included the Prime Minister.

### Surrender of Port Arthur.

The capitulation of Port Arthur was officially announced on Tuesday, and will render the opening of 1905 memorable. After the capture of Sungshushan Fort by the Japanese, General Stössel decided that further resistance was no longer practicable. He accordingly opened negotiations with General Nogi on the afternoon of January 1st under a flag of truce, and the negotiations being favourably received, arrangements for a general act of surrender were speedily concluded. A telegram conveying the news to the people of Tokio provoked striking manifestations of exultation, and in an incredibly short space of time the firing of aerial bombs and daylight rockets began in different quarters. In St. Petersburg the news also created much excitement, and elicited warm expressions of praise for the conspicuous heroism with which General Stössel had conducted the defence. The siege of Port Arthur commenced on February 8th last year. The Mikado expressed high appreciation of the loyalty and endurance displayed by General Stössel in the cause of his country, and a desire that all the honours of war should be extended to him. After the capitulation General Stössel was given permission to send a cablegram to the Tsar declaring that further resistance on the part of the Port Arthur garrison was useless. The essential points of the agreement embodying the conditions of surrender, received at the Japanese Legation, are that the whole fortress, ships, arms, ammunition, military buildings, and other Government property shall be given up; that the Japanese reserve free action when those objects are considered to have been destroyed or injured after the signing of the agreement; that plans of forts, torpedoes, mines, military and naval officers' lists shall be delivered; and that soldiers, sailors, volunteers, and other officials shall be taken prisoners; but, in consideration of the brave defence they have made, military and naval officers and civil officials attached shall be allowed to bear arms, keep private property of immediate necessity of daily life, and also to return to Russia upon parole not to take, till the end of the war, arms in action opposed to Japanese interest.

### Admiral Togo and the Spirits of the Dead.

On Saturday Admiral Togo attended the funeral services at Tokio of a number of officers and men who were killed at Port Arthur while serving him, and pronounced a remarkable eulogy upon them in the course of which he said: "As I stand before your spirits I can hardly express my feelings. Your personality is fresh in my memory, your corporeal existence has ceased, but your passing from the world has been in the gallant discharge of your duty, by virtue of which the enemy's fleet on this side of the world has been completely disabled. Our combined fleet retains the undisputed command of the seas. I trust that this will bring peace and rest to your spirits. It is my agreeable duty to avail myself of the occasion of my presence in this city, whither I have been called by the Emperor, to report our successes to the spirits of those who sacrificed their earthly existence for the attainment of so great a result."

### The Principal Events of 1904.

The first important event of 1904 happened on January 6, when the Russian reply to the Japanese Note was presented. In the same month Mr. Whitaker Wright committed suicide after sentence, and Mrs. Maybrick was released. In February the King opened Parliament, war commenced between Japan and Russia, Prince Alexander of Teck married Princess Alice of Albany, and Dr. Jameson was appointed Premier of Cape Colony. In April the Anglo-French Agreement was signed, the Budget statement was made in the House of Commons, and Illig, Somaliland, was captured by the British. In May Sir William Harcourt made his last speech in Parliament, and the French Ambassador to the Vatican was recalled. In June the Governor of Finland was assassinated. In July the Anglo-German Arbitration Treaty was signed, M. de Plehve was assassinated in St. Petersburg, and a free pardon was conveyed to Mr. Adolph Beck. In August the British Mission reached Lhasa, the Tsaritsa gave birth to a son, and the Archbishop of Canterbury arrived in Quebec. In September an Italian prince was born, and King Peter of Servia was crowned. In October British trawlers were fired upon by the Russian Baltic Fleet, and the Russian Government agreed to British demands. In November Mr. Roosevelt was elected President of the United States for the second time, and the King and Queen of Portugal visited England. In December Russia's last battleship at Port Arthur was disabled, and Admiral Togo was welcomed back to Japan. The deaths of the year included Admiral Sir H. Keppel, the Duke of Cambridge, Sir Edwin Arnold Queen Isabella of Spain, Miss Nellie Farren, Maurus Jokai, Sir H. M. Stanley, Mr. G. F. Watts, ex-President Kruger, M. Waldeck Rousseau, Dean Hole, Prince Herbert Bismarck, Professor Finsen, Sir William Harcourt, the King of Saxony, and Mr. Dan Leno.

### The Watts Exhibition.

Never before has such a comprehensive exhibition been made of the works of Mr. G. F. Watts, R.A., as that which opened at Burlington House on Monday, although both the New Gallery and the Grosvenor have each collected the works of the great artist together once before. The most striking point of the 248 pictures at the Royal Academy is their extraordinary variety and their interest is increased by the fact that they stretch over a period of nearly 70 years. The first picture is a portrait of the artist, painted when he was a stripling of 17; the last is also a portrait of himself, a grand old man of 80 years. His earliest pictures are all of interest, but it was not until about 1860 that his pictures began to assume the characteristics inseparably associated in those days with the name of Watts. The exhibition also contains some of the works of the late Frederick Sandys, and the completed model of the national memorial to Queen Victoria now being executed by Mr. Brock. The central figure, the Queen enthroned, is 13 feet high, and the whole work is on a herculean scale.

### Notes and Queries.

**FOR REGULATIONS SEE PAGE 200.**

*Deodorant.*

(103) Will you kindly tell me of anything that can be burnt in the sick-room of a patient suffering from offensive cancer as a deodorant?—*R. F.*

You can buy scented pastilles to burn, but the best deodorisers are extreme cleanliness and fresh air. Using Sanitas freely, too, should help greatly to purify the atmosphere in such a case.

*Parish Nurse.*

(104) Can you kindly tell me how a nurse can be procured for a very large parish in London? I am afraid a salary cannot be guaranteed, but it would prove very interesting work for a lady with means.—*Z. S.*

You had better advertise.

*Insurance.*

(105) Is there any company in which I could insure against accidents and disease, or disease only? I am a trained nurse.—*E. C D.*

Write to the Secretary of the Royal National Pension Fund for Nurses, 28 Finsbury Pavement, E.C.

*Nurses for India.*

(106) Seeing in THE HOSPITAL that nurses are required for training native women in India, I should feel greatly obliged if you will kindly send me Mrs. Creighton's address.—*F. D.*

See "The Response to the Cry from India" in THE HOSPITAL *Nursing Section* for December 24th. Do not write to Mrs. Creighton.

*Patent.*

(107) I am writing to ask your advice as to how I am to set about getting a provisional patent for an idea of mine. . . ?—*Patricia*

Your idea sounds promising, but before doing anything write to the Patent Office, 25 Southampton Buildings, Chancery Lane, E.C.

*Johannesburg.*

(108) Some time ago I saw a paragraph in THE HOSPITAL about a new maternity hospital in Johannesburg, and should be obliged if you could tell me to whom I could apply for information as to getting a post on the staff.—*A. Z.*

Write to the Matron, Maternity Hospital, Johannesburg.

*Probationer.*

(109) Please say if a person is considered as "employed" while they are at a hospital on trial before being accepted as probationer; that is, are they responsible to mention this under the head of "where previously employed" in filling up an application form for a subsequent place?—*E. S. V.*

When applying for a vacancy as probationer, you must certainly mention if you have been in any other hospital, no matter for how short a time.

*Antiseptic Bath.*

(110) Can you kindly tell me the best way of putting a suppurating compound comminuted fractured tibia in an antiseptic bath when the patient is confined to his bed? The kind of bath used at present does not hold enough lotion to cover the tibia, as if very full the bed gets swamped?—*Sister P.*

It must be impossible to cover the limb in the bath you are using at present, as the leg must be on a splint and would be at such an angle that certainly not more than the foot could be under water. There is an upright bath to be had, but it would not suit your case. The only means of getting such a wound covered seems to be by constant irrigation. Then the bath itself could be dispensed with and the limb lie on a mackintosh, splint and all, with a receiver below for the lotion to drip into.

*Lime in Water.*

(111) The water of this district is strongly impregnated with lime, and the result of drinking it, either alone or in cooking, is constant flatulence and distension. What are the best methods of counteracting these effects and softening the water?—*R. W.*

For cold drinking water try small quantities of carbonate of soda; for washing water, a little borax will act as a softener. But boiled water should have no ill effects, as in boiling the lime is deposited. For treatment of the flatulence and distention, if really severe, a medical man must, of course, be consulted.

**Handbooks for Nurses.**

| | Post Free. |
|---|---|
| "The Nursing Profession: How and Where to Train" ... | 2s. 4d. |
| "The Nurses' Pronouncing Dictionary" ... ... ... | 2s. 0d. |
| "Nursing: its Theory and Practice" (Lewis) ... ... | 3s. 6d. |
| "Surgical Bandaging and Dressings" ... ... ... | 2s. 0d. |
| "A Complete Handbook of Midwifery" (Watson) ... | 6s. 4d. |

Of all booksellers or of The Scientific Press, Limited 28 & 29 Southampton Street, Strand, London, W.C.

### For Reading to the Sick.

"YE ARE MY WITNESSES."

How bright these glorious spirits shine!
Whence all their white array?
How came they to the blissful seats
Of everlasting day?

Lo! these are they from sufferings great
Who came to realms of light;
And in the Blood of Christ have wash'd
Those robes that shine so bright.

*Watts.*

The Apostle tells us that the "cloud of witnesses" compasses us about, that they are about us and around—nay, among us, that they wrap us up as a cloud. Bright and numerous, but, unknown, like the countless stars of the summer night, which the child looks up to, not knowing that they are stars, but thinking them a soft, milk-white cloud. Calm and peaceful are they; their race is done, their work is finished; they are at rest from their labours. Once they toiled in the battlefield of life; once they were tried in the furnace of affliction, or with the soft eating mildew of prosperity; once they perhaps stood in the fires of persecution, and boldly bore their testimony to the name of Christ, and went joyfully to prison or to death for the sake of the Lord they loved. Or, it may be, theirs was the humble lot, the hidden trial, the secret combat day by day with old and cherished sins, the struggle against remorse, that would tell them repentance was too late, or that God's mercy was no more. The constant doing for the sake of Christ of little things against which taste and inclination rebelled; giving up their own way; putting themselves out to help another; denying themselves the possession of some wished-for object, or checking the exhibition of some talent, because experience told them it was a snare to themselves, or Christian tact, that it would hurt another. And through all the petty scenes of mortification, and all the little crosses which make up a modern Christian's martyrdom, they still had faith that stayed upon their God; still felt that, so they bore what Christ sent them, they were in the path of safety, whether God called them to little sufferings or to great; still persevered in the uninteresting round of common things, making love shed a bright light over all their life, and finding in every shadow the shadow of the Cross, still persevering to the end in union with Him who loved them, died for them, and lives in them, even "Jesus, the Author and Finisher of their faith."

*Rev. G. C. Harris.*

Remembering the presence of these unseen witnesses, and conscious of their knowledge in some way of what is taking place here below—although we have no assurance that they could even hear us, if we tried to speak to them—we have to "run with patience the race that is set before us, looking unto Jesus," their Lord and our Lord: "looking unto Jesus, the Author and Finisher of our faith," Who begins our life of faith in this world, and completes it in His Paradise.

*Bishop Webb.*

A Good Man, and an Angel! these between
How thin the barrier! What divides their fate?
Perhaps a moment or perhaps a year.
Angels are men in lighter habit clad
Nor are our brothers thoughtless of their kin;
Yet absent but not absent from their love.

*Young.*

